# Comprehensive Engineering Performance Evaluation of the IRRAflow Active Fluid Exchange System with IRRAS Bolt for Intracranial Fluid Management

**DOI:** 10.7759/cureus.100435

**Published:** 2025-12-30

**Authors:** Behnam Rezai Jahromi, Riam Badr, Dave Asbury, John Unser

**Affiliations:** 1 Neurosurgery, Helsinki University Hospital, Helsinki, FIN; 2 Engineering, IRRAS Corporation, San Diego, USA; 3 Strategic Initiatives, IRRAS Corporation, San Diego, USA

**Keywords:** active fluid exchange, cerebrospinal fluid (csf) dynamics, chronic subdural hematoma (csdh), external ventricular drain (evd), intracranial bolt, intracranial pressure (icp) management, intraventricular hemorrhage (ivh), irraflow, neuro critical care, subarachnoid hemorrhage (sah)

## Abstract

The IRRA*flow* Active Fluid Exchange System (IRRAS, San Diego, California, USA) is a novel medical device designed to manage intracranial pressure (ICP) and cerebrospinal fluid (CSF) in patients with neurological conditions such as subarachnoid hemorrhage (SAH), intraventricular hemorrhage (IVH), and chronic subdural hematoma (cSDH). This study presents a series of evaluations assessing the system’s performance across multiple technical aspects: catheter-bolt sealing, catheter liquid flow characterization with bolt fixation, torque limit evaluation of the metal bolt and plastic wing interface, fluid equilibrium, injection volume accuracy, 24-hour pressure drift evaluation of IRRA*flow* ICP sensor output, and pressure accuracy. The results are reported relative to predefined acceptance criteria for each test. The engineering and potential clinical implications of these findings are described in the context of intracranial fluid management.

## Introduction

Effective management of intracranial pressure (ICP) and cerebrospinal fluid (CSF) dynamics is critical in treating neurological conditions such as subarachnoid hemorrhage (SAH), intraventricular hemorrhage (IVH), and chronic subdural hematoma (cSDH). Elevated ICP, often caused by hydrocephalus, brain swelling, or hematoma, can lead to secondary brain injury and poor functional outcomes [1]. In SAH, for instance, hydrocephalus occurs in approximately 30% of patients, contributing to intracranial hypertension and increased mortality [2]. Similarly, IVH requires rapid CSF management to facilitate clot clearance and prevent post-hemorrhagic hydrocephalus, which is associated with prolonged exposure to blood breakdown products [3-7]. cSDH, common in elderly patients, benefits from efficient drainage to reduce reoperation rates [8].

The IRRA*flow* Active Fluid Exchange System (IRRAS, San Diego, California, USA) was developed to integrate active fluid exchange and ICP monitoring. Unlike traditional external ventricular drains (EVDs), which are prone to occlusion in over 40% of cases, IRRA*flow*’s dual-lumen catheter and automated irrigation system are intended to manage fluid dynamics through controlled irrigation and passive drainage [9, 10]. The IRRAS Bolt is designed to provide secure cranial access, enabling the safe insertion and stable placement of the IRRA*flow* catheter for CSF management. This study consolidates technical evaluations, including three IRRAS Bolt evaluations and four IRRA*flow* system-level evaluations, to assess the system’s performance and characterize performance parameters relevant to its use in neurocritical care settings.

## Technical report

Methods

Seven independent evaluations were conducted to test specific performance characteristics of the IRRA*flow* system. Each evaluation employed standardized methodologies, with detailed procedures described in the respective results sections. 

The IRRAS Bolt (ICB010) evaluations included catheter-bolt sealing, which tested pressure integrity at -185 mmHg and +185 mmHg; catheter liquid flow characterization with bolt fixation, which monitored long-term flow consistency; and torque limit evaluation of the metal bolt and plastic wing interface.

The IRRA*flow* system evaluations included fluid equilibrium, which tested the system’s ability to maintain stable fluid dynamics; injection volume accuracy, which evaluated the precision of fluid bolus delivery; a 24-hour pressure drift evaluation of IRRA*flow* ICP sensor output; and pressure accuracy, which assessed ICP monitoring precision across a range of pressures.

All tests used IRRA*flow* components, including control units, catheters, and fluid test chambers (IRRAS), with measurements recorded using calibrated equipment.

Catheter-Bolt Sealing Methods

Experimental design: The IRRA*flow* bolt sealing was evaluated by sealing the catheter in the bolt, applying pressures of -185 mmHg and +185 mmHg using a pressure transducer simulator [11]. With the catheter secured in the IRRAS Bolt, the assembly was evaluated for pressure integrity under negative (-185 mmHg) and positive (+185 mmHg) conditions for 60 seconds, in accordance with NS28 standard requirements, representing the specified test extremes.

Equipment: Two IRRAS Bolts (Lot: 2410-2050); one Delta-Cal™ Transducer Simulator and Tester (Asset No: 0291); one stopwatch (Asset No: 0048).

Procedure: The catheter was inserted into the bolt, and the bolt cap was tightened to secure and seal the catheter. A negative or positive pressure was then applied. Initial and final pressures were recorded after 60 seconds, with three trials performed per condition.

Catheter Liquid Flow Characterization with Bolt Fixation Methods

Experimental design: The catheter-bolt system was tested by securing the catheter to the IRRAS Bolt. Irrigation and drainage flow rates were measured over a 29-day period. Performance was evaluated by applying controlled pressure for both irrigation and drainage, with daily measurements collected to assess system consistency and reliability.

Equipment: Two IRRAS Bolts (Lot: 2410-2050); two catheters with attached four-way stopcocks (7001286); two bolt assemblies (10FR, 7001302); one IRRA*flow* Control Unit; one balance scale (minimum precision 0.01 g, capacity 2200 g); one ruler; one stopwatch; one syringe; and one modified tubing (7000813).

Procedure: The catheter was inserted through the bolt, extending 5 cm beyond the distal end, and secured by tightening the bolt cap. Initial irrigation and drainage flow rates were measured without securing the bolt to establish baseline values. After securing the bolt, the tests were repeated, and daily measurements were recorded for 29 days. Flow rates were calculated based on the mass of fluid delivered and drained.

Bolt Torque Characterization Methods

Experimental design: Torque testing of the IRRAS Bolt was performed using a custom fixture and a digital torque meter. The IRRAS Bolt was secured in the fixture, which was then clamped in a bench vice. A torque meter was used to apply rotational force in both directions, and the corresponding torque values were recorded.

Equipment: Three IRRAS Bolts; one digital torque meter (Asset No: 0086); one bench vice; one torque adapter assembly (7000623).

**Figure 1:**
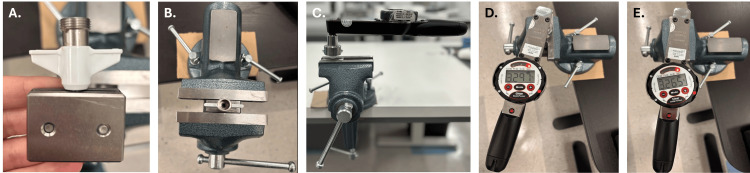
Experimental procedure for bolt torque characterization (A) The bolt was inserted into a vice with a torque adapter assembly. (B) The fixture containing the IRRAS Bolt was clamped securely in a bench vice. (C) A digital torque meter was attached to the bolt head. (D) The torque meter was rotated clockwise until it reached at least 3.160 N·m, and the peak torque value was recorded. (E) The torque meter was rotated counterclockwise until it reached at least 3.160 N·m, and the peak torque value was recorded. Image credits: Riam Badr (Author)

Procedure: Torque characterization began by inserting the IRRAS Bolt into the torque adapter assembly (Figure 1A). The fixture containing the bolt was then clamped securely in a bench vice (Figure 1B). A digital torque meter was attached to the bolt head (Figure 1C). The torque meter was rotated clockwise until it reached at least 3.160 N·m, and the peak torque value was recorded (Figure 1D). The torque meter was then rotated counterclockwise until it reached at least 3.160 N·m, and the peak torque value was recorded (Figure 1E).

Fluid Equilibrium Methods

Experimental design: Tests were conducted using the IRRA*flow* system connected to a simulated “Pseudo Brain” test chamber. Under clinical conditions, ICP within the ventricles depends on the height of the drainage collection system (DCS010). This test was designed to quantify two key variables affecting fluid equilibrium: (1) drainage collection system height (independent variable) and (2) the resulting ICP within the test chamber (Pseudo Brain), recorded as the dependent variable.

Equipment: One IRRA*flow* Control Unit (ICCU-020); one fluid test chamber (custom-made to simulate the cranial environment); one calibrated balance scale (for measuring mass as a proxy for fluid volume).

Procedure: The test chamber was filled with an initial volume of 500 mL of water, and the catheter was primed to eliminate air from the catheter and tubing. The IRRA*flow* system was set to an irrigation bolus rate of 30 mL/hour, selected as an average between the available irrigation rates. Measurements of mass and pressure were recorded during treatment cycles consisting of measurement, drainage, and irrigation states. Tests were performed across drainage collection system heights (-10 to +10 mmHg) and chamber pressures (+5 to +10 mmHg).

Injection Volume Accuracy Methods

Experimental design: This study evaluated the IRRA*flow *system’s accuracy in delivering 1 mL bolus injections across baseline pressures (-10, 0, 10, 20, 50, and 100 mmHg) and chamber volumes (100 to 500 mL).

Equipment: Three IRRA*flow* Control Units (ICCU-020); three IRRA*flow* Catheters (ICAT 030); one fluid test chamber (“Pseudo Brain,” P/N: 7001476); one calibrated balance scale (Asset No: 0336).

Procedure: The fluid chamber was prepared by filling it with 100 mL of water and connecting it to the IRRA*flow* system via fluid-sealing adapters (Appendix A). The tube set and IRRA*flow* catheter were then primed to eliminate air bubbles. Testing was performed by initiating bolus injections at each pressure setting, with three measurements recorded for each condition. The mass of injected fluid was measured to determine accuracy.

For analysis, the acceptance criterion specified that the average volume of three boluses must be 1.0 ± 0.4 mL.

Twenty-Four-Hour Pressure Drift Evaluation of IRRAflow ICP Sensor Output Methods

Experimental design: To evaluate long-term pressure stability, a 24-hour pressure drift test was conducted on the IRRA*flow* Active Fluid Exchange System. Pressure drift is the gradual shift in a sensor’s reading over time at a constant pressure (for these experiments, pressure is set to 10 mmHg and measured by the measurement of a direct, fluid-coupled Deltran® Disposable Pressure Transducer). Testing was performed using a custom-built fluid test chamber, referred to as the “Pseudo Brain,” designed to simulate intracranial fluid dynamics, including physiological pressure and compliance. The IRRA*flow* device was operated in accordance with its instructions for use (IFU) and evaluated against a calibrated reference water column reservoir set to 10 mmHg.

Pressure data were continuously acquired using a BIOPAC MP160 data acquisition system (BIOPAC Systems, Inc., Goleta, California, USA) paired with AcqKnowledge® software version 5.0.8.1 (BIOPAC Systems, Inc.). The primary objective was to quantify the deviation in pressure between the IRRA*flow* system and the static reference pressure over a 24-hour period. Both the IRRA*flow* and reference transducers were zeroed at the start of the test, and the pressure difference was calculated over time to assess drift performance. All test units were evaluated under identical environmental and fluidic conditions to ensure data comparability.

Equipment: Three IRRA*flow* Control Units (ICCU-020); three IRRA*flow* Catheters (ICAT030); three IRRA*flow* Tube Sets (ICDS020); one Deltran® Disposable Pressure Transducer (Model DPT‑100) used as the reference transducer; three IRRA*flow* Transducer Modules connected to the BIOPAC MP160 system via adapters for test unit measurements; one DELTA-CAL™ Pressure Transducer Simulator and Tester; and one BIOPAC MP160 Data Acquisition System with AcqKnowledge® Software version 5.0.8.1.

Procedure: The pressure drift test began once the IRRA*flow* system was primed and placed into operation mode per the IFU. The BIOPAC hardware was assembled and connected to both the fluid test chamber and the pressure transducers. Room temperature and the water bath temperature were maintained throughout the test. The reference water reservoir was adjusted and verified to remain at a constant 10 mmHg baseline.

Prior to data collection, the AcqKnowledge® software was configured to autosave the data file and display real-time pressure graphs for each test channel. Data acquisition was then initiated and allowed to run continuously for 24 hours. The reference transducer and the IRRA*flow* sensor were zeroed to 0 mmHg at t = 0; the deviation between the reference and IRRA*flow* pressures represents drift.

Pressure Accuracy Methods

Experimental design: The IRRA*flow* system was tested across baseline pressures ranging from -10 to 100 mmHg. Measurements were obtained using a Deltran® Disposable Pressure Transducer (Model DPT‑100; Utah Medical Products, Inc., West Midvale, Utah, USA) as the reference transducer and IRRA*flow* Transducer Modules connected to the BIOPAC system via adapters to the IRRA*flow* Control Unit. Pressure readings were displayed on the bedside monitor. Testing was performed with the fluid test chamber set to physiologically relevant compliance levels to evaluate pressure accuracy.

Equipment: Three IRRA*flow* Control Units (ICCU-020); one Deltran® Disposable Pressure Transducer (Model DPT‑100) for reference pressure measurements; three IRRA*flow *Transducer Modules with BIOPAC adapters for test unit measurements; one fluid test chamber simulating a cranial environment; one water-filled syringe with shutoff valve for applying target pressures.

Procedure: The test setup involved connecting a water-filled syringe to the fluid test chamber. Target pressures were applied in increments of -10, 0, 10, 20, 50, and 100 mmHg. After each pressure adjustment, a minimum of 20 seconds was allowed for stabilization, after which pressure was monitored for one minute and then recorded. Each pressure level was measured three times. Accuracy was defined as a maximum difference of ±2 mmHg or ±10% between the reference pressure and the test pressure.

Note: Given the descriptive nature of this technical evaluation, this study is underpowered for formal inferential analysis.

Results

Catheter-Bolt Sealing (Pressure Decay)

With the catheter secured in the IRRAS Bolt, the assembly was evaluated for pressure integrity under negative (-185 mmHg) and positive (+185 mmHg) conditions for 60 seconds, in accordance with NS28 standard requirements and worst-case scenario conditions. Pressure decay was measured using the DELTA-CAL™ Pressure Transducer Simulator (Utah Medical Products, Inc.), with each condition tested in triplicate. The acceptance criterion was a pressure change within ±10%. 

Findings: At -185 mmHg, the average final pressure was -184 mmHg (0.54% and 0.72% difference for bolts 1 and 2, respectively), and at +185 mmHg, the average final pressure was +183 mmHg (-1.26% and -0.90% difference for bolts 1 and 2, respectively). Both samples (IRRAS Bolt 1 and 2) (Tables 1, 2) met the acceptance criterion, indicating that the catheter-bolt interface maintained pressure within the predefined limits.

**Table 1:** IRRAS Bolt 1 sealing (pressure decay)

Set Pressure (mmHg)	Average Final Pressure (mmHg)	Percent Difference	Acceptance Criteria	Meet Acceptance Criteria?
–185	-184	0.5%	± 10%	Pass
+185	+183	-1.3%	± 10%	Pass

**Table 2:** IRRAS Bolt 2 sealing (pressure decay)

Set Pressure (mmHg)	Average Final Pressure (mmHg)	Percent Difference	Acceptance Criteria	Meet Acceptance Criteria?
–185	-184	0.7%	± 10%	Pass
+185	+183	-0.9%	± 10%	Pass

The IRRAS Bolt with a secured catheter maintained pressure within the predefined acceptance criterion of ±10%, demonstrating that the catheter-bolt sealing mechanism met the specified pressure decay criterion under both negative and positive pressures.

Minor discrepancies between the initial and final pressure readings remained within the acceptable range and were within the predefined tolerance range. These results indicate that the bolt provides a seal around the catheter that is sufficient to maintain pressure within the specified tolerance range. Effective sealing is critical for accurate ICP management in the therapeutic treatment of neurological conditions such as intraventricular hemorrhage, subarachnoid hemorrhage, ventriculitis, and traumatic brain injury.

Clinical importance: Catheter-bolt sealing ensures that the IRRA*flow* system maintains pressure integrity at applied pressures of -185 mmHg and +185 mmHg, which is important for precise ICP management in IVH, SAH, ventriculitis, and cSDH. In IVH and SAH, elevated ICP caused by blood accumulation or hydrocephalus can lead to secondary brain injury, and any leakage or pressure loss could compromise therapeutic outcomes [1]. In ventriculitis, maintaining a sealed system prevents contamination, reducing the risk of infection exacerbation [12]. In cSDH, stable pressure control supports consistent drainage, minimizing the risk of reoperation [8].

This study demonstrated that pressure decay remained within ±10%, confirming the system’s reliability. These results underscore the importance of effective sealing for accurate ICP monitoring and safe fluid management, both of which are essential to preventing complications such as herniation and infection.

Catheter Liquid Flow Characterization with Bolt Fixation

Irrigation and drainage flow rates were measured daily over 29 days using two catheters secured in the IRRAS Bolt. The setup included the IRRA*flow* control unit and a calibrated balance scale.

**Figure 2:**
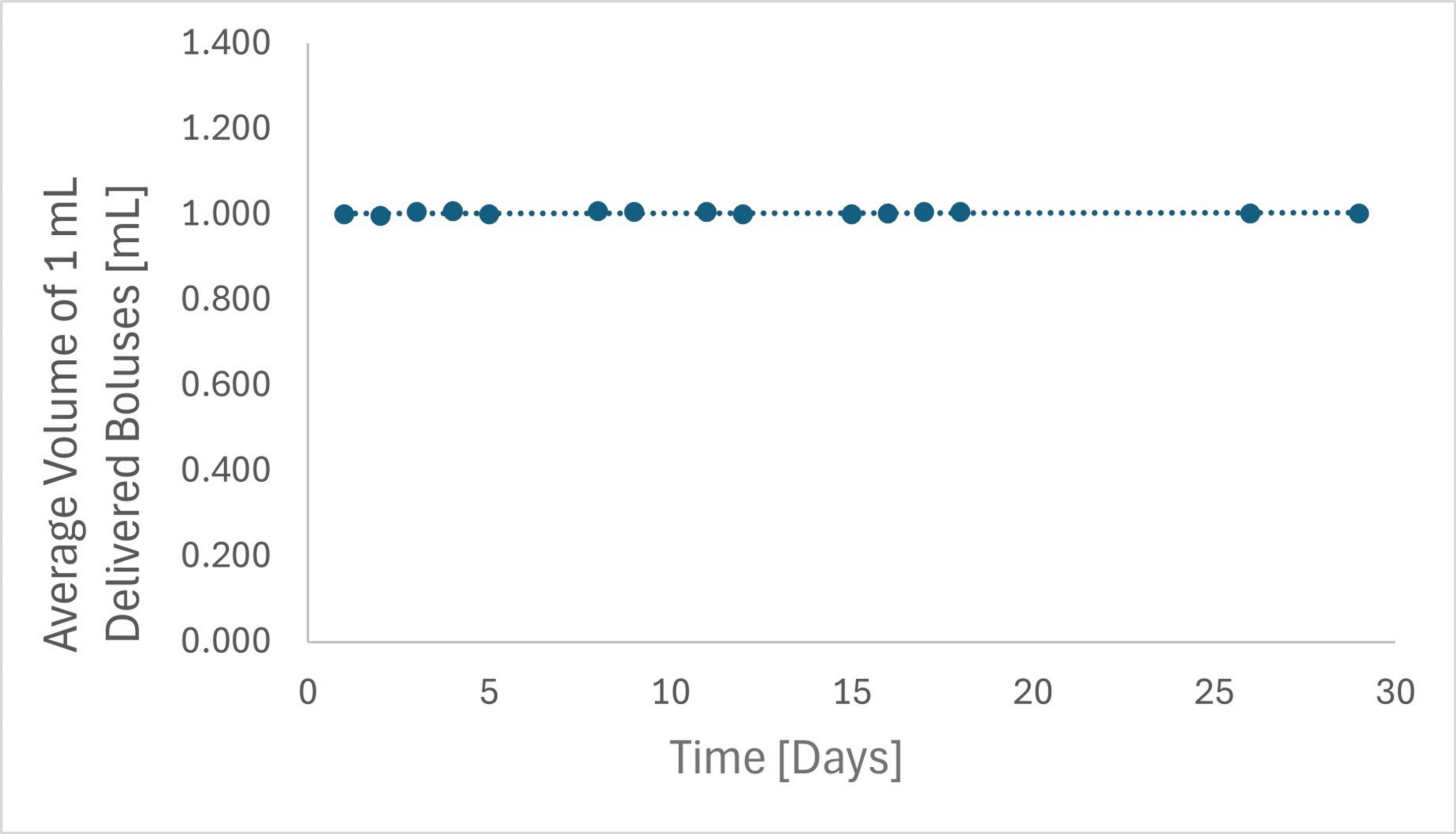
Representative irrigation flow performance data of the IRRAflow Catheter secured in the IRRAS Bolt, illustrating irrigation flow rates over a 29-day testing period.

Findings: Irrigation flow rates (Figure 2, Table 3) were reported to two decimal places and averaged 1.004 ± 0.004 mL/bolus, showing minimal variation. Irrigation boluses were delivered three times daily per bolt; therefore, the mean daily irrigation volume was 3.011 ± 0.011 mL across all days and both bolts. The average bolus volume remained consistent at 1.004 ± 0.004 mL throughout the study.

**Table 3:** Irrigation flow performance

Time (Days)	Average Volume/Bolus (mL)	Notes on Irrigation Flow Rate
0-29	1.004 ± 0.004	Consistent

**Figure 3:**
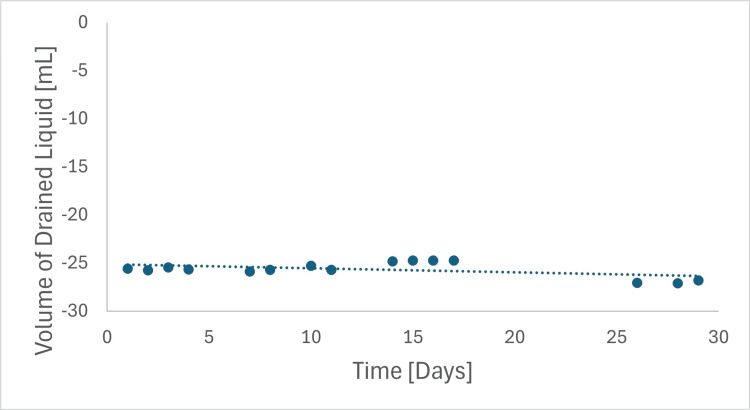
Representative flow performance data of the IRRAflow Catheter secured in the IRRAS Bolt, illustrating drainage flow rates over a 29-day testing period.

The drainage flow rate averaged -25.09 ± 0.83 mL/min, with minimal variation (Figure 3, Table 4). The pressure difference between the water inlet and outlet was 15 ± 2 cmH₂O. A slight increasing trend in flow rate was observed over the 29-day period, likely attributable to relaxation of the inner lumen diameter, thereby reducing flow resistance. Importantly, bolt securement did not impede flow, and flow remained within the range observed for the non-bolted condition.

**Table 4:** Drainage flow performance

Time (Days)	Average Drainage Flow Rate (mL/min)	Notes on Drainage Flow Rate
0-29	-25.09 ± 0.83	Stable and Reproducible

Overall, the test demonstrated consistent irrigation and drainage performance throughout the 29-day evaluation, with irrigation boluses delivered precisely and drainage flow rates remaining stable. Negative values represent measured outflow.

The irrigation and drainage flow tests showed consistent results across both samples. The average irrigation flow rate remained at 1.004 mL, with no significant variations, indicating that the bolt’s design did not measurably reduce irrigation flow relative to baseline measurements.

The minimal variation in flow rates between samples indicates that the bolt’s mechanical design, incorporating both sealing and securing mechanisms, stabilizes the catheter without producing detectable changes in irrigation or drainage flow rate within the resolution of this test.

Overall, these findings demonstrate stable irrigation and drainage performance over the 29-day test interval under the specified conditions.

Clinical importance: Consistent irrigation and drainage flow rates over 29 days (irrigation: 1.004 ± 0.004 mL; drainage: -25.09 ± 0.83 mL) are relevant for long-term management of IVH, SAH, ventriculitis, and cSDH. In IVH, reliable flow prevents catheter occlusion, which occurs in over 40% of traditional EVDs, ensuring continuous clot clearance as presented in previous literature [9]. In SAH, consistent irrigation reduces blood breakdown products, lowering vasospasm risk [13]. In ventriculitis, stable flow supports prolonged antibiotic delivery and CSF clearance, critical for infection control [12]. In cSDH, consistent drainage reduces hematoma re-accumulation, decreasing reoperation rates [8]. 

Bolt Torque Characterization

The torque applied to the IRRAS Bolt plastic wings tested the ruggedness of the device. The acceptance criterion required that the coupling of the injection-molded wings and the bolt withstand a minimum of 3.160 N·m of torque without failure, ensuring safe insertion and removal of the product.

Findings: The torque test results demonstrated that all tested over-molded bolts (10 FR, PN 7001301) met the minimum acceptance criterion of 3.160 N·m in both clockwise and counterclockwise directions. Torque values in both directions exceeded the threshold, with all individual bolts passing under standard loading conditions (Table 5). These findings indicate that the coupling between the injection-molded wings and the bolt maintained mechanical integrity under typical torque applications. No failures or separations were observed during standard bolt testing under the tested torque conditions. 

**Table 5:** Measured clockwise (CW) and counterclockwise (CCW) torque values for IRRAS Bolts

Bolt	Applied Clockwise Torque ≥ 3.160 (N⋅m)	Applied Counterclockwise Torque ≥ 3.160 (N⋅m)	Acceptance Criteria	Result (CW)	Result (CCW)
1	X	X	≥ 3.160	Pass	Pass
2	X	X		Pass	Pass
3	X	X		Pass	Pass

In the ultimate torque test, each bolt was subjected to progressively higher torque until failure (Table 6). All samples withstood torque levels well above the minimum requirement of 3.160 N·m. In two of the three samples, failure occurred at the bolt itself, which fractured under elevated loads. Importantly, the injection-molded wings and their interface with the bolt remained intact, with no evidence of mechanical separation. This indicates that the over-molding process provides a mechanically stable bond, and such bolt fractures should not occur under normal clinical use. 

**Table 6:** Ultimate torque values for IRRAS Bolts

Bolt	Applied Torque (N⋅m)	Observation	Image	Acceptance Criteria	Result
1	7.618	No breakage - Bolt was deformed and slipping under the torque load	A	The coupling between the injection-molded wings and the bolt shall withstand a minimum torque of 3.160 N⋅m without mechanical failure.	Pass
2	9.241	Breakage at the bolt	B		Pass
3	9.410	Breakage at the bolt	C		Pass

**Figure 4:**
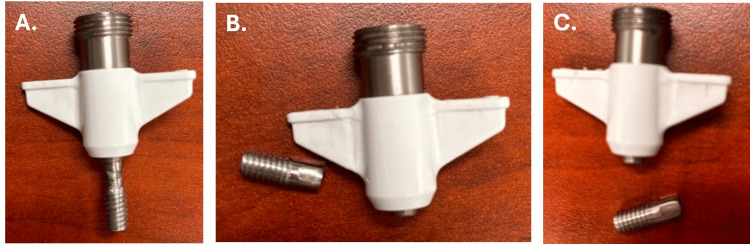
IRRAS Bolts post ultimate torque test Image credits: Riam Badr (Author)

The results demonstrate that the wing-to-bolt coupling withstood torque levels exceeding the minimum specified requirement under test conditions. Under ultimate testing, the bolt may fracture under excessive torque, while the wings remain intact, indicating that the failure mode under extreme torque was localized to the bolt rather than the wing-to-bolt interface (Figure 4).

Clinical importance: A rugged cranial bolt with integrated wings is relevant to ensure secure fixation and to prevent component separation when high torque is applied during catheter insertion or removal. The mechanical integrity of the bolt-wing interface under clinically relevant torque loads is essential, as failure at this junction could complicate catheter placement or removal. Mechanical testing demonstrated that the IRRA*flow* bolt tolerates ≥3.160 N·m of applied torque without evidence of damage, indicating a margin between the minimum specified torque requirement and the ultimate torque to failure, and exhibits structural failure only at extreme torques >7 N·m, well beyond those required for insertion and removal.

Fluid Equilibrium

**Figure 5:**
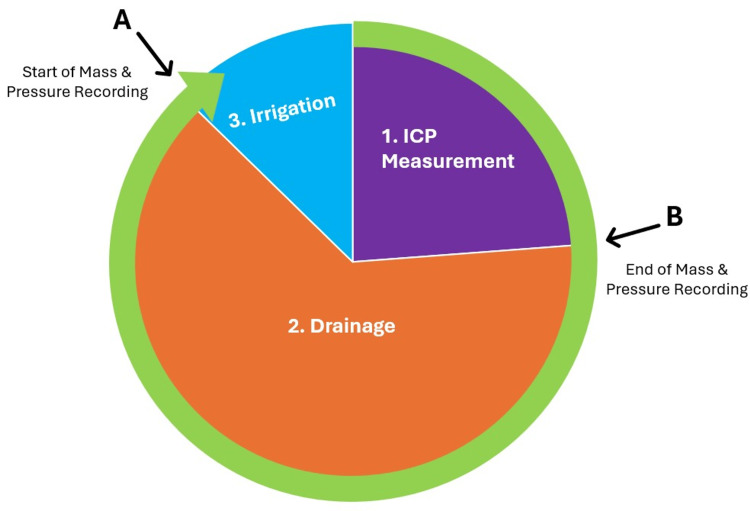
IRRAflow control unit’s treatment cycle The treatment cycle begins with the ICP measurement state (purple), proceeds to the drainage state (orange), and concludes with the irrigation state (blue). This cycle repeats until interrupted. Mass and test chamber pressure are recorded before each Irrigation state and after each ICP measurement state, with these points indicated by black arrows. ICP: intracranial pressure

Fluid equilibrium was tested using a “Pseudo Brain” test chamber, with mass and pressure recorded during treatment cycles (measurement, drainage, irrigation) (Figure 5) at varying pressures (-10 to +10 mmHg drainage; +5 to +10 mmHg chamber).

**Figure 6:**
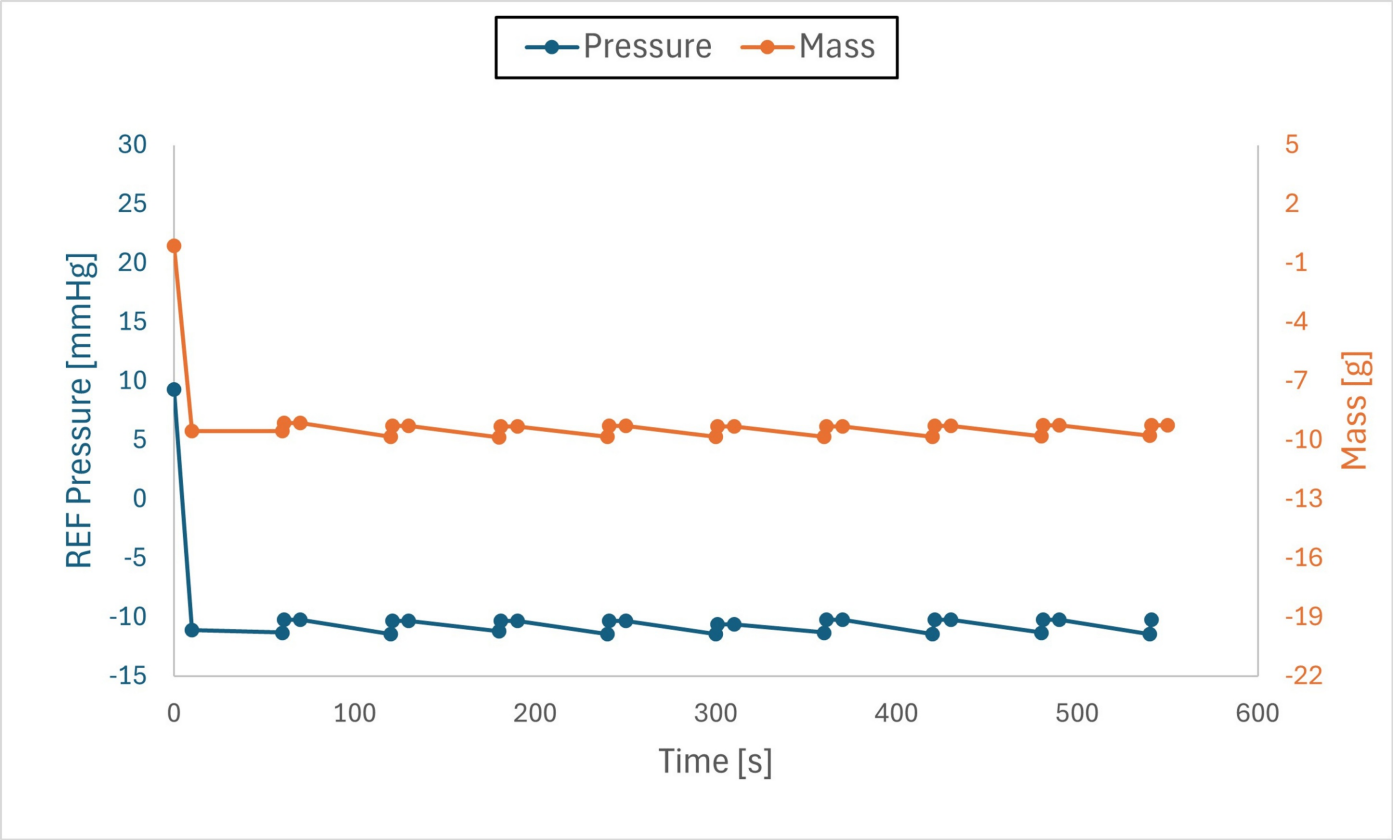
Pressure and mass vs. time (initial pressure set to +10 mmHg and drainage collection system set to –10 mmHg) This figure shows pressure and mass versus time during fluid exchange, with the initial test chamber pressure set to +10 mmHg and the drainage collection system (DCS010) set to –10 mmHg. Pressure (blue) and mass (orange) were recorded at defined intervals (before each irrigation cycle and after each intracranial pressure (ICP) measurement event). Both parameters declined rapidly at the onset of the test to the DCS010 set pressure. Fluid equilibrium was achieved once the test chamber pressure matched the DCS010 pressure of –10 mmHg.

**Figure 7:**
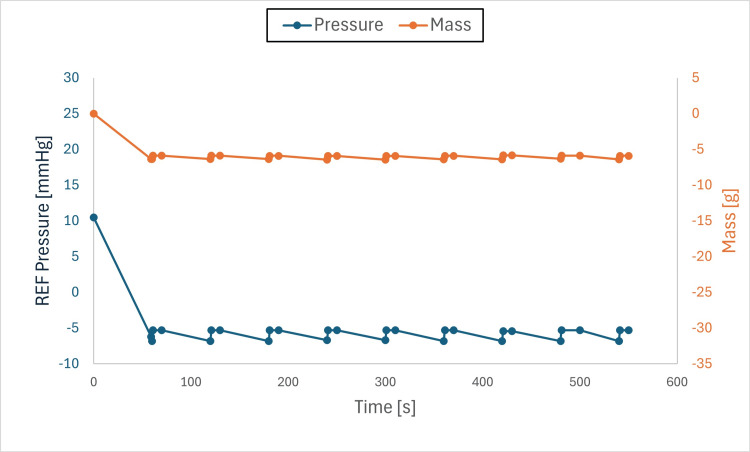
Pressure and mass vs. time (initial pressure set to +10 mmHg, drainage collection system set to –5 mmHg) This figure shows pressure and mass versus time during fluid exchange, with the initial test chamber pressure set to +10 mmHg and the drainage collection system (DCS010) set to –5 mmHg. Pressure (blue) and mass (orange) were recorded at defined intervals (before each irrigation cycle and after each intracranial pressure (ICP) measurement event). Both parameters declined rapidly at the onset of the test to the DCS010 set pressure. Fluid equilibrium was achieved once the test chamber pressure matched the DCS010 pressure of –5 mmHg.

**Figure 8:**
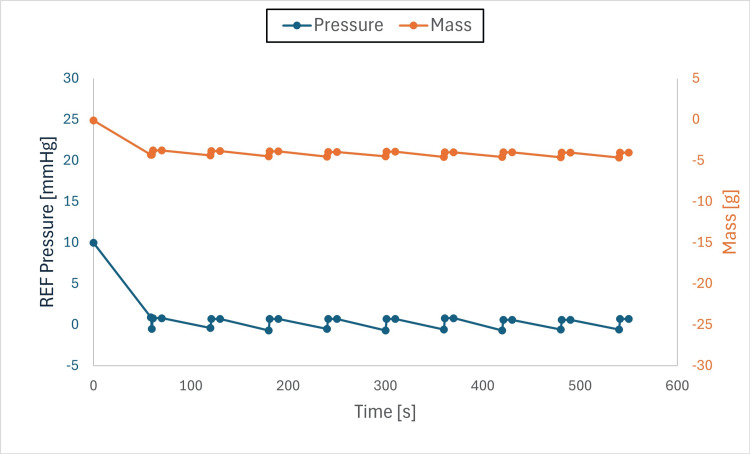
Pressure and mass vs. time (initial pressure set to +10 mmHg, drainage collection system set to 0 mmHg) This figure shows pressure and mass versus time during fluid exchange, with the initial test chamber pressure set to +10 mmHg and the drainage collection system (DCS010) set to 0 mmHg. Pressure (blue) and mass (orange) were recorded at defined intervals (before each irrigation cycle and after each intracranial pressure (ICP) measurement event). Both parameters declined rapidly at the onset of the test to the DCS010 set pressure. Fluid equilibrium was achieved once the test chamber pressure matched the DCS010 pressure of 0 mmHg.

**Figure 9:**
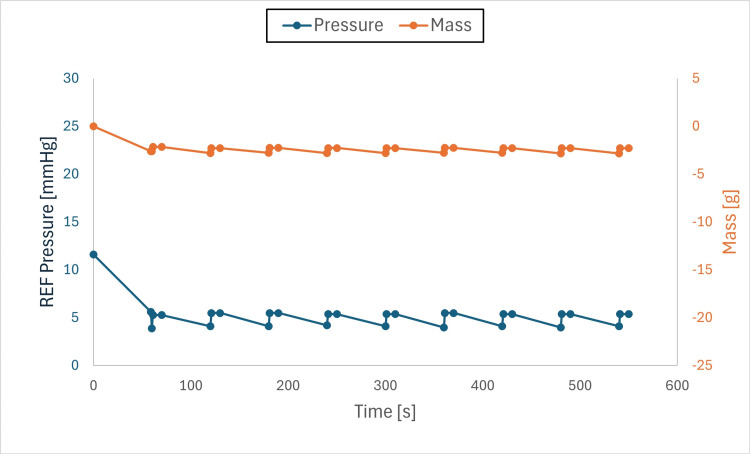
Pressure and mass vs. time (initial pressure set to +10mmHg, drainage collection system set to +5mmHg) This figure shows pressure and mass versus time during fluid exchange, with the initial test chamber pressure set to +10 mmHg and the drainage collection system (DCS010) set to +5 mmHg. Pressure (blue) and mass (orange) were recorded at defined intervals (before each irrigation cycle and after each intracranial pressure (ICP) measurement event). Both parameters declined rapidly at the onset of the test to the DCS010 set pressure. Fluid equilibrium was achieved once the test chamber pressure matched the DCS010 pressure of +5 mmHg.

**Figure 10:**
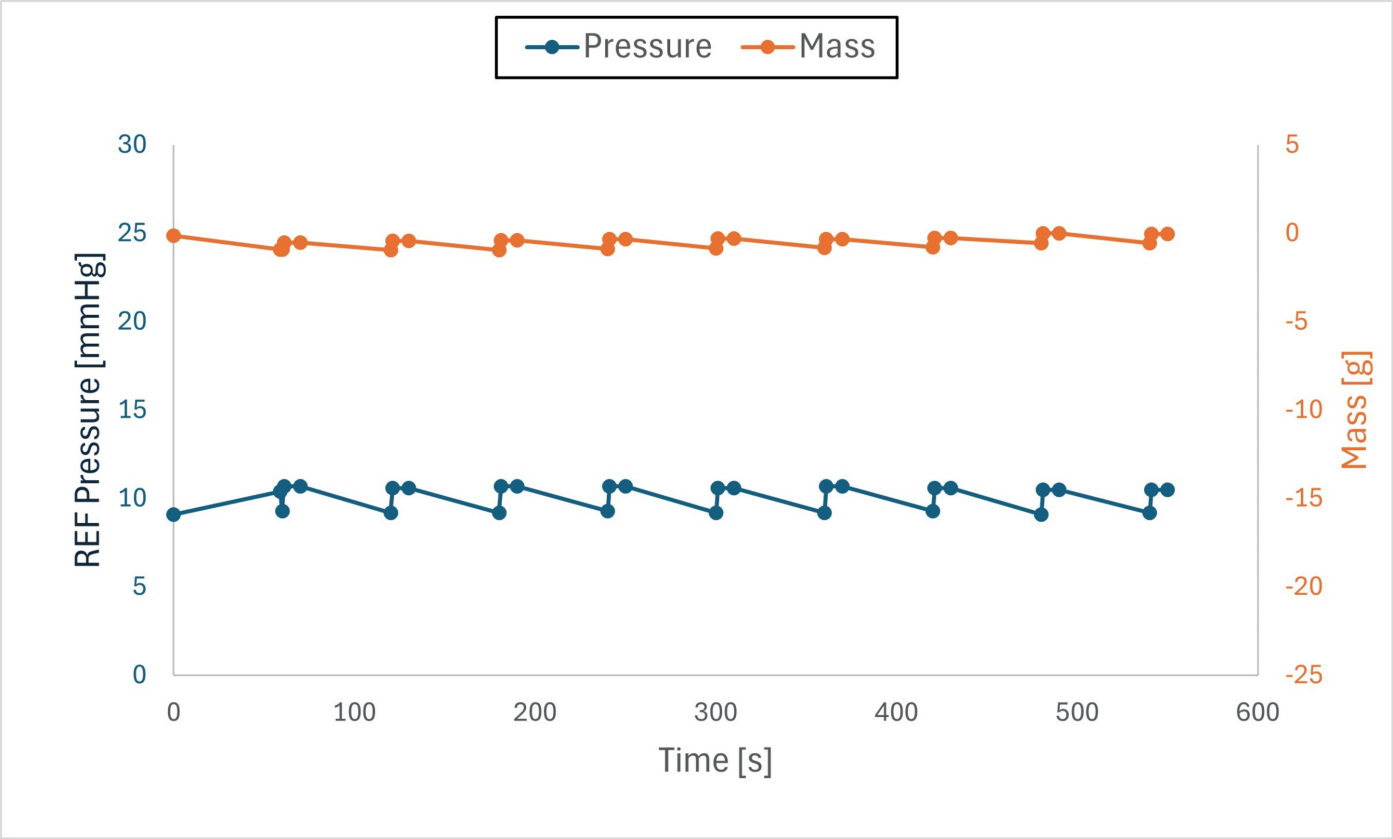
Pressure and mass vs. time (initial pressure set to +10 mmHg, drainage collection system set to +10 mmHg) This figure shows pressure and mass versus time during fluid exchange, with the initial test chamber pressure set to +10 mmHg and the drainage collection system (DCS010) set to +10 mmHg. Pressure (blue) and mass (orange) were recorded at defined intervals (before each irrigation cycle and after each intracranial pressure (ICP) measurement event). Both parameters remained stable at 0.00 g, with only slight fluctuations over time, as fluid equilibrium was reached at the onset of the test when the test chamber pressure equaled the DCS010 pressure of +10 mmHg.

**Figure 11:**
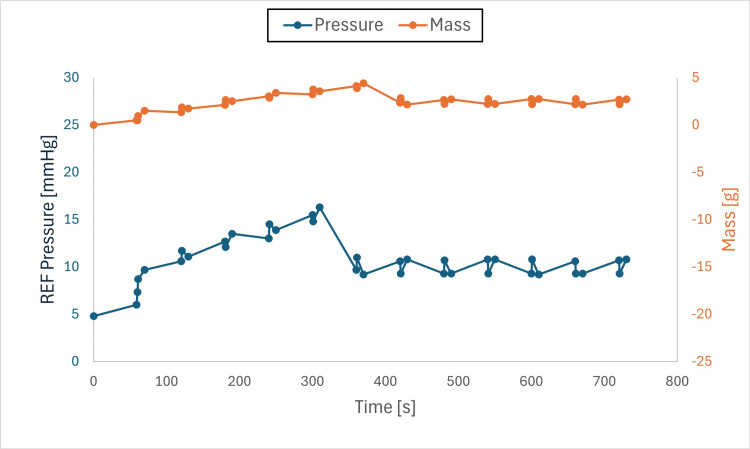
Pressure and mass vs. time (initial pressure set to +5mmHg, drainage collection system set to +10mmHg) This figure shows pressure and mass versus time during fluid exchange, with the initial test chamber pressure set to +5 mmHg and the drainage collection system (DCS010) set to +10 mmHg. Pressure (blue) and mass (orange) were recorded at defined intervals (before each irrigation cycle and after each intracranial pressure (ICP) measurement event). At the onset of the test, both parameters gradually increased to approximately +16 mmHg, higher than the DCS010 set pressure (see Discussion), before decreasing until fluid equilibrium was reached at +10 mmHg, when the test chamber pressure matched the DCS010 pressure. Pressure and mass then stabilized at the DCS010 set pressure.

Findings: The system stabilized both mass and pressure, achieving equilibrium when chamber and drainage pressures were aligned. Pressure adjustments produced transient spikes; however, equilibrium was re-established, and brief air-gap spikes (Figures 6-11) did not compromise overall performance.

Observations

Pressure gradients: Fluid equilibrium was reached when the test chamber pressure matched the drainage system pressure.

Air gaps: Instances of transient pressure spikes were observed due to the presence of an air gap as liquid entered the drainage collection system, resulting in a temporary pressure increase. Once the liquid filled the tube, the pressure stabilized.

When the DCS010 was elevated above the test chamber (Figure 11), the resulting liquid column exerted hydrostatic pressure on the chamber. This caused a fluid gap to form at the entry point of the drainage tube. To overcome the height of this column and allow flow, the chamber pressure initially had to rise. If the tube outlet had been positioned at or near the intended drip height, pressure would have stabilized at 10 mmHg. However, due to the additional vertical height of the tubing and the presence of an air gap, chamber pressure rose to 16 mmHg before gradually decreasing and stabilizing at 10 mmHg, the pressure corresponding to the final height of the drainage collection system.

**Figure 12:**
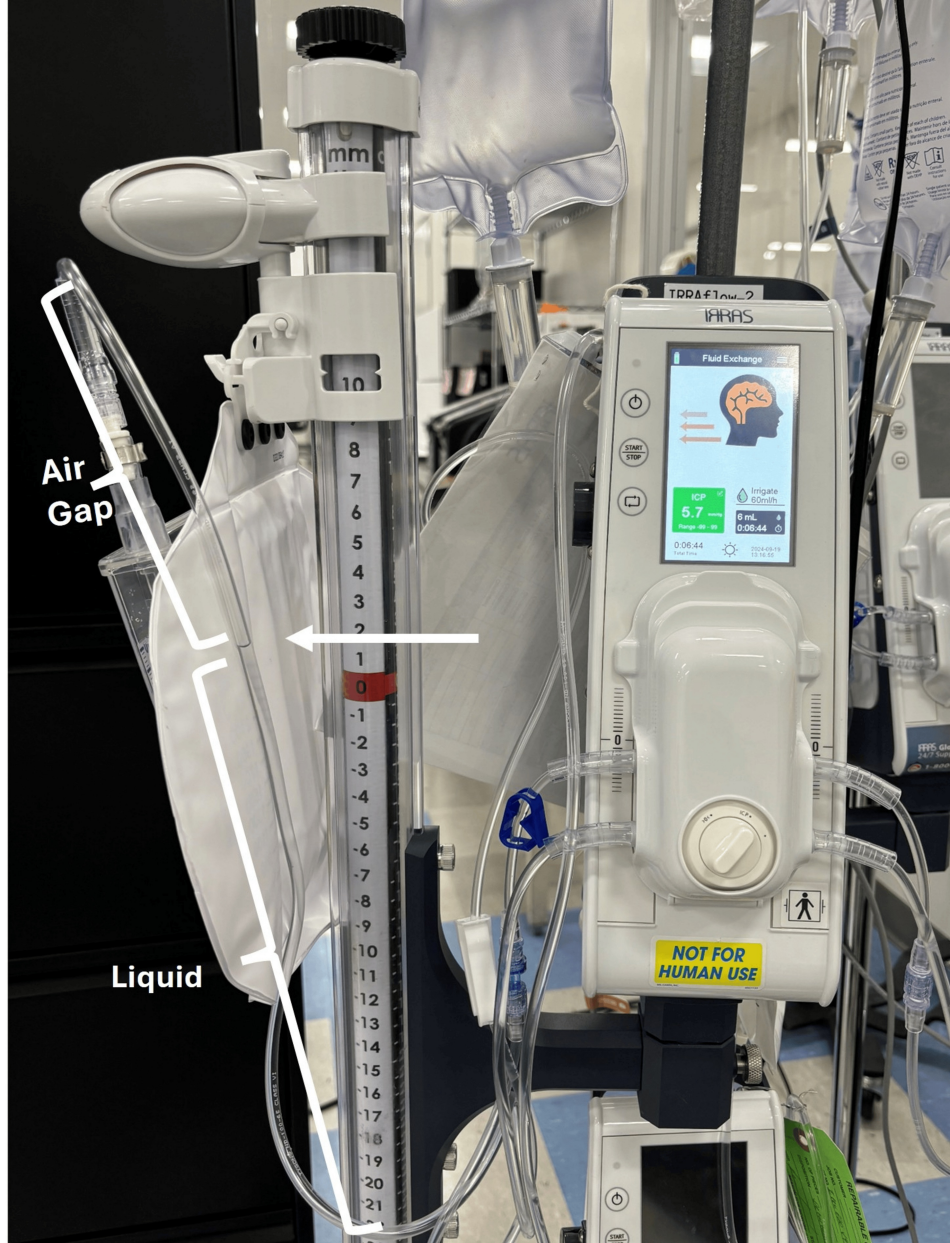
Representative figure of the air gap within the IRRAflow drainage tube Image credits: Riam Badr (Author)

The transient increase in pressure occurred because of the air gap within the tubing loop (Figure 12). This loop added +6 mmHg to the DCS010 set pressure, resulting in a total of 16 mmHg (10 mmHg + 6 mmHg = 16 mmHg). As expected, once the loop was completely filled with liquid, the chamber pressure equalized to the DCS010 set pressure of 10 mmHg.

In all conditions, the system responded to pressure gradients and stabilized, achieving IRRA*flow* fluid equilibrium.

The IRRA*flow* system successfully maintained fluid dynamics stability within a closed system, even under varying pressure gradients. The relationship between mass and pressure was directly proportional, with pressure increasing as mass increased and vice versa. Initial fluctuations were attributed to the system adjusting its fluid balance to reach the target pressure, a necessary step to ensure overall stability and accuracy.

When the drainage collection system (DCS010) liquid column pressure was lower than the test chamber pressure, both pressure and mass decreased as fluid exited the chamber until equilibrium was reached. When the liquid column pressure equaled the test chamber pressure, mass and pressure remained stable, confirming the system’s ability to maintain equilibrium under these conditions.

When the DCS010 liquid column pressure was higher than the test chamber pressure, both pressure and mass increased as fluid was pushed into the chamber until pressures equalized. This transition was consistent with the system’s dynamic adjustments, although an unexpected rise in pressure occurred due to the formation of an air gap in the tubing. The air gap, combined with the added vertical height of the tubing, temporarily elevated chamber pressure until the gap was filled with liquid, at which point pressure stabilized at the set level.

Another variable not directly tested in this study was fluid viscosity. Higher viscosity would be expected to reduce drainage capacity and may increase fluid retention. Clinicians should consider this factor when setting drainage collection system height, as it may contribute to higher retained volumes. Notably, CSF production is approximately 25 mL/h, whereas under minimal pressure differential, the system demonstrated a flow rate of 25 mL/min (60× greater flow capacity than required to drain the hourly CSF production volume). As irrigation increases around the catheter, fluid viscosity would be expected to decrease, emphasizing the importance of closely monitoring drainage collection system height when using the IRRA*flow* system.

These findings identify air-gap formation and tubing height as variables that can transiently affect pressure until the system re-equilibrates. Despite these effects, the IRRA*flow* system transitioned between measurement, drainage, and irrigation states and restored equilibrium at the drainage collection system set pressure under all tested conditions.

Clinical importance: Maintaining fluid equilibrium is important for stabilizing ICP and CSF dynamics in IVH, SAH, ventriculitis, and cSDH. This study demonstrated that the IRRA*flow* system achieved equilibrium when chamber and drainage pressures aligned, despite minor transient spikes from air gaps. In IVH, equilibrium supports consistent clot clearance without over-drainage, reducing the risk of hydrocephalus [3]. For SAH, it stabilizes ICP to help prevent secondary injury [1]. In ventriculitis, equilibrium facilitates infection control by maintaining steady CSF flow [12]. In cSDH, it minimizes hematoma re-accumulation, lowering reoperation rates [8].

The system’s ability to adapt to varying pressures and account for fluid viscosity confirms reliable performance, though physicians must closely monitor drainage collection system height to optimize outcomes. This is especially critical in patients with low compliance, where excessive fluid retention can cause disproportionate increases in ICP.

Injection Volume Accuracy

The system’s ability to deliver 1 mL bolus injections was tested across baseline pressures (-10 to 100 mmHg) and fluid chamber volumes (100-500 mL). The acceptance criterion required an average bolus volume of 1.0 ± 0.4 mL.

**Figure 13:**
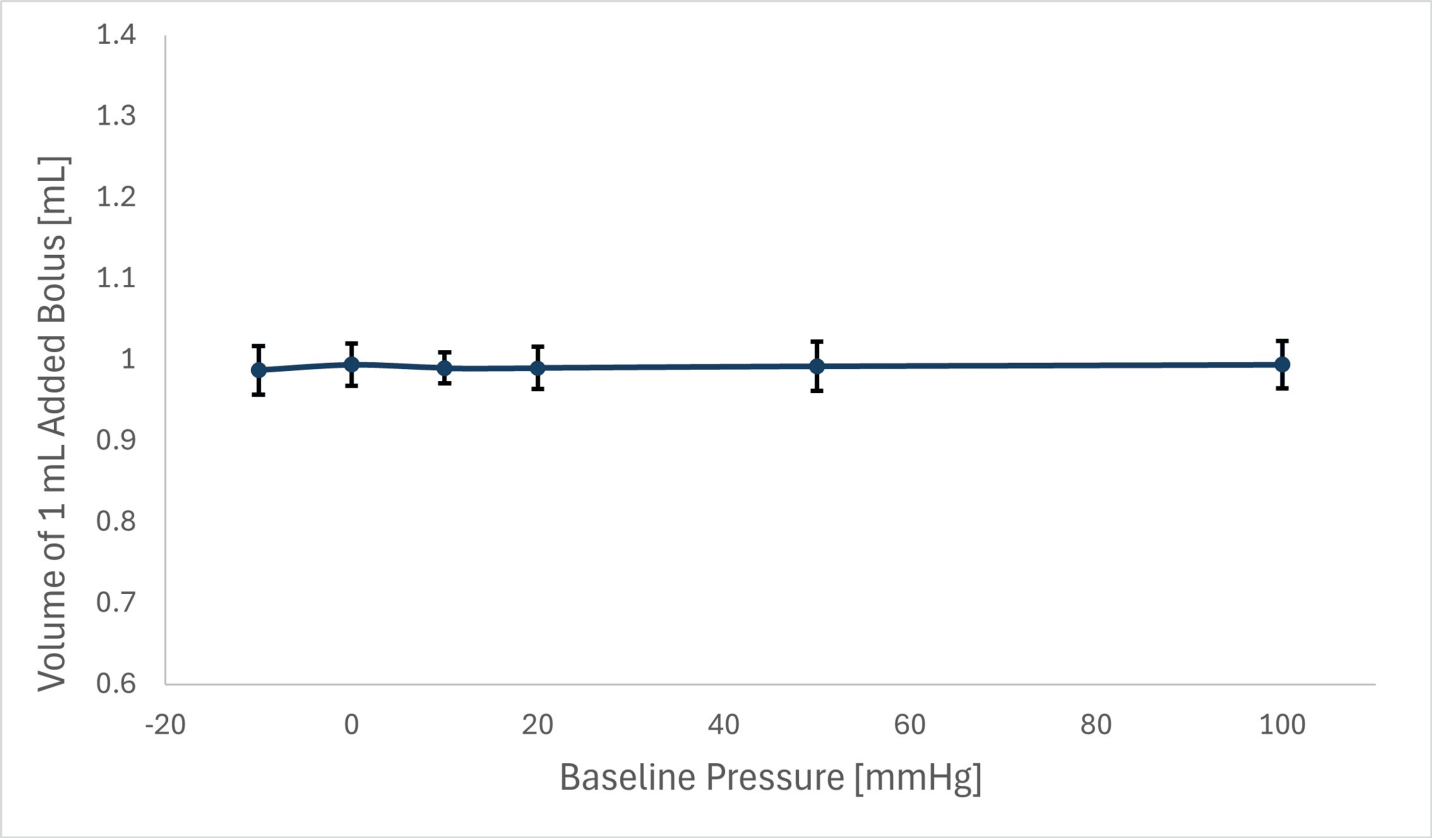
Accuracy of added bolus volume across pressures in IRRAflow system This figure illustrates the relationship between baseline pressure (mmHg) and injected bolus volume (mL), with error bars indicating variability (1-standard deviation equal to 0.027 mL).

Findings: The mean delivered volume was 0.991 ± 0.027 mL across all conditions, consistently meeting the accuracy criterion of 1.0 ± 0.4 mL/bolus. Variability was minimal, and no consistent trend or pressure-dependent variation in delivered bolus volume was observed across chamber pressures tested from −10 to 100 mmHg (Table 7, Figure 13).

**Table 7:** Bolus accuracy across baseline pressures

Pressure (mmHg)	Min (mL)	Max (mL)	Mean ± SD (mL)	Meets Acceptance Criteria?
-10	0.90	1.07	0.987 ± 0.030	Pass
0	0.94	1.07	0.994 ± 0.026	Pass
10	0.96	1.03	0.990 ± 0.019	Pass
20	0.94	1.05	0.990 ± 0.026	Pass
50	0.91	1.10	0.992 ± 0.030	Pass
100	0.94	1.07	0.994 ± 0.029	Pass
All pressures	0.91	1.10	0.991 ± 0.027	Pass

The IRRA*flow* system demonstrated repeatable performance in delivering accurate bolus volumes across a wide physiological pressure range. Proper priming to remove air bubbles is essential, as trapped air may result in under-measurement of bolus volume. In addition, the system’s safety mechanisms limited high-pressure conditions within the framework of the tested pressure range.

Clinical importance: The IRRA*flow* system’s ability to deliver precise 1 mL bolus injections (0.991 ± 0.027 mL) is relevant for controlled irrigation in neurocritical care. In IVH, accurate bolus delivery supports clot lysis by administering thrombolytics or saline to break down hematomas, thereby reducing the risk of hydrocephalus [3]. For SAH, precise irrigation aids in clearing blood products, lowering the incidence of vasospasm and delayed cerebral ischemia [13]. In ventriculitis, controlled fluid exchange enables effective antibiotic delivery and CSF clearance without excessive pressure fluctuations [12]. In cSDH, accurate irrigation minimizes hematoma recurrence by facilitating debris clearance and reducing the likelihood of reoperation [8]. The system’s precision ensures safe and effective fluid management, which is critical for therapeutic success in neurocritical care.

Twenty-Four-Hour Pressure Drift Evaluation of IRRAflow ICP Sensor Output

The pressure drift evaluation was conducted over a 24-hour period using two independent pressure transducers: the IRRA*flow* system and a reference channel. The IRRA*flow* pressure output was compared to the reference pressure transducer. Pressure transducers were directly coupled to the thermally stabilized, constant-pressure chamber set to 10 mmHg, representing the expected physiological ICP under stable conditions. Per the test protocol, the acceptable drift range was defined as ±1 mmHg, corresponding to a set pressure range of 9.0 to 11.0 mmHg.

**Figure 14:**
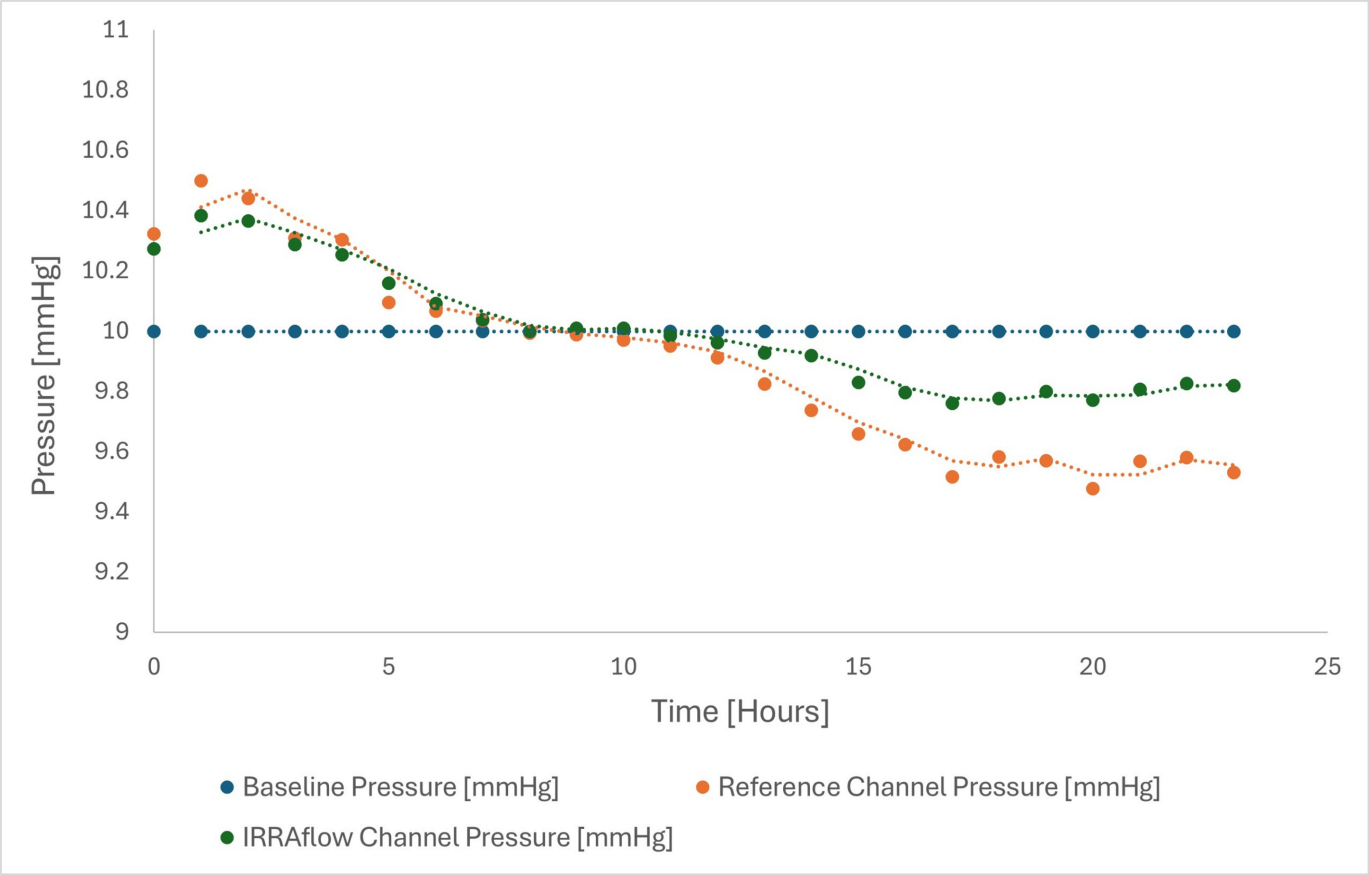
Pressure drift over time Comparison of IRRAflow and reference channels against baseline pressure (mmHg).

Findings: The baseline pressure remained constant at 10 mmHg throughout the test duration (Figure 14). The IRRA*flow* and reference channels were monitored continuously, and hourly averages were calculated to assess gradual drift behavior. The IRRA*flow* channel pressure ranged from approximately 9.7 to 10.3 mmHg, showing a slight downward trend over time. The reference transducer exhibited mild drift, ranging from 10.5 mmHg at the start to approximately 9.4 mmHg by hour 24, consistent with expected variability in baseline measurements during extended monitoring.

Despite these shifts, both channels consistently remained within the ±1 mmHg acceptance criterion throughout the test duration. This demonstrates that the pressure-sensing performance of both transducers, including the IRRA*flow* system, remained within the predefined drift tolerance over the full 24-hour monitoring window, thereby meeting the requirements for intracranial pressure drift tolerance in clinical and simulated-use environments.

Clinical importance: Accurate measurement of ICP is essential to guide therapy in neurocritical care and, in the case of the IRRA*flow* system, to enable the safe delivery of drainage, irrigation, and intraventricular drug infusion. The system demonstrated stable and reliable ICP monitoring with a measured drift of <1.0 mmHg over 24 hours. To maintain long-term accuracy, the IRRA*flow* software requires re-zeroing of the pressure transducer every 24 hours. This is accomplished by rotating the intelligent cassette knob to the “>0<” position, which simultaneously resets the zero point and restarts the 24-hour timer. This process ensures consistent alignment of the transducer with atmospheric reference and supports accurate, reliable pressure monitoring throughout therapy.

Pressure Accuracy

Pressure accuracy was assessed across set pressures (-10 to 100 mmHg) using a simulated fluid test chamber and BIOPAC transducers. The acceptance criterion was defined as ±2 mmHg or ±10%, whichever was greater.

Findings: The system maintained accuracy within the specified limits at all tested pressures, with all measurements falling within ±2 mmHg or ±10% of the setpoint (whichever was greater) (Table 8). The largest discrepancy was 3.47%, observed at a 100 mmHg set pressure, which still met the acceptance criterion. Minor variations were likely due to slight non-linearities in the pressure transducers; however, all results remained within both the transducer’s accuracy specification and the requirements for neurological pressure monitoring.

**Table 8:** Assessment of pressure accuracy across varying set pressures in the IRRAflow system. Table summarizes the pressure accuracy results. All measured values met the acceptance criterion of ±2 mmHg or ±10%, whichever was greater.

Set Pressure (mmHg)	Δ IRRA*flow* Pressure (mmHg)	Meets Acceptance Criteria?
-10	+0.81 ± 0.83	Pass
0	-0.29 ± 1.24	Pass
10	-0.33 ± 0.52	Pass
20	-1.00 ± 0.64	Pass
50	-1.99 ± 1.08	Pass
100	-3.36 ± 1.75	Pass

The IRRA*flow* system demonstrated consistent pressure accuracy across all tested conditions. Although minor discrepancies were observed, particularly at higher pressures, all measurements remained within the predefined acceptance criterion.

These differences may be attributed to calibration variations and pressure transducer scaling. Overall, the system’s performance met the predefined pressure accuracy criterion across the tested range.

Clinical importance: Accurate ICP monitoring is vital for managing IVH, SAH, ventriculitis, and cSDH, as small deviations can lead to catastrophic outcomes such as brain herniation or inadequate treatment. This study demonstrated that the IRRA*flow* system maintained pressure accuracy within ±2 mmHg or ±10% across -10 to 100 mmHg, which is within the specified accuracy limits of the test configuration. In SAH, precise ICP control reduces mortality associated with intracranial hypertension [1]. For IVH, accurate pressure readings guide clot clearance and help prevent post-hemorrhagic hydrocephalus [2]. In ventriculitis, precise monitoring enables effective CSF drainage without over- or under-drainage, which could worsen infection or cause ventricular collapse [12]. In cSDH, accurate pressure management supports optimal drainage, lowering recurrence rates [8].

The system’s reliability allows physicians to make informed decisions regarding drainage and irrigation settings, directly supporting safer and more effective neurocritical care.

## Discussion

This technical report contrasts with the article “Reliability and performance of the IRRA*flow* system for intracranial lavage and evacuation of hematomas-A technical note” [14]. While that paper raised concerns regarding the system’s reliability and performance, its conclusions were drawn from constrained test conditions, which did not reflect the true engineering performance or clinical use of the IRRA*flow* system. The present study examines additional performance characteristics by performing a broader set of evaluations under standardized, physiologically relevant conditions.

The findings reported by Haldrup et al. (2024) [14] were based on an ex vivo setup using the Silverline® F10 bolt and an earlier IRRA*flow* software version (v3.0), which differs substantially from the currently released software (v4.0) and hardware configuration evaluated in our study. Their observed catheter occlusion, impaired irrigation flow, and pressure fluctuations were associated with compression of the dual-lumen catheter inside a non-indicated, non-IRRAS bolt, a configuration not recommended in the IFU. In contrast, our evaluations used the IRRAS-designed bolt and the current IRRA*flow* system architecture, which prevents the catheter compression mechanism observed in the Haldrup setup.

Furthermore, Haldrup’s reported pressure artifacts may have been influenced by their use of a reference sensor positioned independently of the IRRA*flow* catheter tip, as suggested by the pressure recordings reported during their drift assessment [14]. These conditions differ from the clinical configuration and do not incorporate the updated sensor-drainage algorithms implemented in software v4.0.

Importantly, none of the failure modes described by Haldrup (bolt-induced occlusion, irrigation obstruction, or persistent pressure instability) were reproduced in our testing under the specified conditions [14]. This suggests that their findings reflect earlier device configurations and off-label use conditions, whereas our results describe the current IRRA*flow* platform under appropriate, indicated use.

The present study was designed to expand upon those observations by performing a broader set of evaluations under standardized, physiologically relevant conditions. The objective of this report is not only to counter the earlier claims but also to provide a more rigorous and comprehensive dataset demonstrating the system’s sealing integrity, flow stability, mechanical durability, pressure accuracy, and long-term reliability.

This technical report evaluates the IRRA*flow* Active Fluid Exchange System with the IRRAS Bolt through seven independent assessments, focusing on engineering performance in intracranial fluid management for conditions such as SAH, IVH, ventriculitis, and cSDH. The evaluations included three bolt-specific tests (catheter-bolt sealing, liquid flow characterization with bolt fixation, and bolt torque characterization) and four system-level tests (fluid equilibrium, injection volume accuracy, 24-hour pressure drift, and pressure accuracy).

Across all assessments, the system met or exceeded predefined acceptance criteria, demonstrating sealing within the specified pressure decay limit, flow performance within the measured ranges, torque capacity above the minimum specification, fluid equilibrium behavior consistent with hydrostatic pressure differences, bolus delivery within the defined volume tolerance, drift within the specified limit, and pressure measurements within the predefined accuracy criterion. These findings provide engineering data that may be relevant when comparing active fluid exchange systems to traditional EVDs, which have reported occlusion rates >40% [15].

The catheter-bolt sealing evaluation tested pressure integrity under extreme conditions (±185 mmHg), revealing minimal decay (average 0.54% at -185 mmHg and -1.08% at +185 mmHg), well within the ±10% acceptance criterion. These results confirm effective sealing, which is essential to prevent leaks that could compromise ICP control or introduce infection risks. Clinically, this reliability supports safe management of IVH and SAH, where blood accumulation elevates ICP and increases the risk of secondary injury [1, 3, 16], and in ventriculitis, where a sealed system minimizes contamination [17]. Evidence from studies such as the CLEAR-IVH (Clot Lysis: Evaluating Accelerated Resolution of Intraventricular Hemorrhage) trial underscores the need for stable drainage systems to facilitate clot clearance without complications [2]; the IRRA*flow* system’s sealing performance aligns with these requirements and may help reduce infection rates, which occur in up to 20% of EVD cases [18].

In the catheter liquid flow characterization with bolt fixation, irrigation accuracy (1.004 ± 0.004 mL/bolus) and drainage outflow capacity (-25.09 ± 0.83 mL/min) remained stable over 29 days. Comparison with the non-bolted catheter group (-30.65 ± 0.03 mL/min) showed no impedance from bolt securement, with a t-test indicating no statistically significant difference in maximum drainage flow rate capacity (t = 9.37, p = 0.011). This long-term consistency addresses EVD occlusion issues, enabling prolonged therapy and supporting reliable treatment outcomes. Clinically, stable irrigation and drainage are critical across multiple conditions. In IVH, reliable flow promotes continuous clot lysis, reducing hydrocephalus risk and ICU stays [3, 9, 16]. In SAH, sustained irrigation facilitates clearance of blood products, mitigating vasospasm [19]. In ventriculitis, stable exchange supports effective antibiotic delivery [18]. In cSDH, consistent drainage reduces hematoma re-accumulation, with studies reporting IRRA*flow* reoperation rates as low as 6.3% compared to higher rates with passive drains [20]. Recent propensity-matched analyses corroborate these findings, demonstrating superior hematoma resolution with active irrigation [20].

The bolt torque characterization confirmed that the metal bolt-plastic wing interface withstood ≥3.160 N·m without failure, with ultimate failure occurring only at >7 N·m, far exceeding clinical requirements. This durability ensures secure cranial fixation, reliable removal, and stable insertion of the bolt relative to the skull. In practice, this robustness may reduce procedural complications in SAH and IVH, where stable access is critical for extended monitoring [1, 2, 16]. Furthermore, clinical data from high-volume centers highlight the burden of EVD malposition, reported in up to 15% of cases [17]. The IRRA*flow* system’s reinforced design mitigates this risk, aligning with guidelines that emphasize secure fixation as essential for optimal outcomes [21].

Fluid equilibrium testing using a Pseudo Brain chamber showed that the system stabilized both mass (drainage outflow volume) and pressure once the pressure differential between the Pseudo Brain and the drainage collection system reached 0. This occurred when chamber pressure equaled the hydrostatic pressure created by the height difference between the chamber and the drainage collection system. Equilibrium was consistently achieved across -10 to +10 mmHg, characterizing the relationship between drainage collection system height and chamber pressure under the tested conditions. Clinically, this is significant for preventing over- or under-drainage in IVH (reducing hydrocephalus) [16, 22], SAH (stabilizing ICP) [1], ventriculitis (supporting balanced clearance) [18], and cSDH (avoiding collapse) [20]. Findings from the EARLYDRAIN (Outcome After Early Lumbar CSF-Drainage in Aneurysmal SAH) trial, which demonstrated reduced vasospasm through RBC removal (~35%) with lumbar drains [19], suggest that IRRA*flow*’s higher irrigation capacity could further enhance clot clearance. Ongoing trials such as ARCH (Active Removal of IntraCerebral Hematoma Via Active Irrigation of the Ventricular System) and VASH may provide comparative data.

Evaluation of injection volume accuracy (conducted without the IRRAS Bolt) demonstrated precise 1 mL bolus delivery (0.991 ± 0.027 mL) across -10 to 100 mmHg and 100-500 mL chamber volumes, meeting the predefined 1.0 ± 0.4 mL acceptance criterion. This precision supports controlled irrigation with thrombolytics or saline. Clinically, accurate bolus delivery accelerates clot resolution in IVH [23], reduces delayed cerebral ischemia in SAH [19], ensures targeted antibiotic dosing in ventriculitis [18], and effectively clears debris in cSDH [20]. Recent studies on automated irrigation support these findings, reporting faster hematoma evacuation [3, 14], directly reflecting the benefits of IRRA*flow*’s accuracy.

The 24-hour pressure drift evaluation showed deviations of <1 mmHg, well within the ±1 mmHg acceptance limit. Re-zeroing every 24 hours maintains long-term stability and ensures alignment of the transducer with the atmospheric reference. This capability supports continuous ICP monitoring, essential for guiding therapy in SAH (reducing mortality) [1] and IVH (preventing hydrocephalus) [2]. Clinical evidence indicates that drift in standard transducers can contribute to mismanagement [24]; IRRA*flow*’s minimal drift performance aligns with consensus standards for neurological monitoring devices [24].

Pressure accuracy testing confirmed readings within ±2 mmHg or ±10% across -10 to 100 mmHg, indicating that the system met the predefined accuracy criterion. This precision is critical for detecting subtle ICP changes in ventriculitis [18] and cSDH [20]. The test model set chamber compliance to 0.7 mL/mmHg, consistent with normal adult values, ensuring physiological relevance and supporting patient-specific adjustments [25, 26].

These findings are consistent with emerging clinical data reporting reduced reoperation in cSDH [20] and improved clot clearance in IVH [3, 27] when active fluid exchange strategies are used. Future applications may include integration with AI-driven predictive analytics for real-time ICP adjustments, expansion to pediatric and traumatic brain injury populations, and randomized trials comparing outcomes against standard EVDs. Additionally, investigation of higher irrigation rates in SAH could further reduce vasospasm, as suggested by EARLYDRAIN [19]. Long-term registries and cost-effectiveness studies will be essential to better understand the clinical and economic impact of different CSF management strategies.

## Conclusions

This comprehensive evaluation presents engineering performance data for the IRRA*flow* Active Fluid Exchange System in the context of ICP and CSF management. Its performance across sealing, pressure regulation, volume delivery, flow stability, compliance, and equilibrium tests is summarized relative to predefined acceptance criteria and discussed with respect to potential clinical applications in SAH, IVH, and cSDH. Integration of the IRRAS Bolt provides secure and durable cranial access, as assessed by torque and sealing evaluations in this study. Further comparative clinical studies are required to quantify differences between this system and traditional EVDs. Further clinical studies are warranted to evaluate the impact of these engineering performance characteristics on patient outcomes.

## Figures and Tables

**Figure 15:**
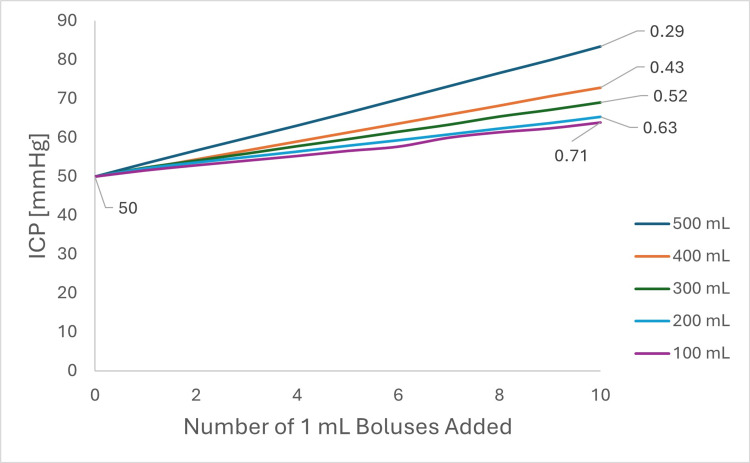
Progression of intracranial pressure (ICP) with varying fluid chamber volumes at 50 mmHg baseline pressure

## Appendices

Appendix A

Fluid Chamber Compliance Methods

Experimental design: The fluid test chamber (IRRAS 7001476) was used to simulate intracranial conditions. Tests were performed across baseline pressures ranging from -10 to 100 mmHg and chamber volumes from 100 to 500 mL. The chamber was incrementally infused with 1 mL boluses, and pressure responses were recorded to calculate compliance.

Equipment: One IRRA*flow* Control Unit (ICCU-020); one fluid test chamber for simulating cranial conditions (IRRAS 7001476); one calibrated Uline Balance Scale (resolution 0.1 g, calibrated per standard).

Procedure: The chamber was filled with water to defined initial volumes, and pressures were adjusted using an external supply. Successive 1 mL boluses were infused, with pressure and mass recorded after each infusion. Compliance was calculated as the change in volume per unit change in pressure (ΔV/ΔP).

Fluid chamber Compliance Results

A fluid test chamber (“Pseudo Brain”) was used to simulate intracranial conditions. A critical step involved evaluating the compliance of the chamber under simulated intracranial pressures and volumes.

Compliance (ΔV/ΔP) was assessed across baseline pressures ranging from -10 to 100 mmHg and initial chamber volumes from 100 to 500 mL. Water was infused in 1 mL boluses, and the resulting pressure changes were recorded to calculate compliance.

Findings: Compliance decreased with increasing chamber volume and baseline pressure. For example, at an initial volume of 100 mL, compliance measured 0.86 mL/mmHg at 0 mmHg and decreased to 0.71  mL/mmHg at 50 mmHg. At 500 mL, compliance measured 0.32 mL/mmHg at 0 mmHg and decreased to 0.29 mL/mmHg at 50 mmHg. At 300 mL, compliance aligned with adult clinical values (0.7 mL/mmHg), indicating that this volume produced a compliance value within the expected adult physiological range.

Compliance measurements across initial volumes and baseline pressures demonstrated the system’s responsiveness to fluid infusions (Table 9). The results confirm that compliance decreases as chamber volume and baseline pressure increase, consistent with the known inverse relationship between compliance and pressure increment per bolus.

**Table 9:** Compliance of the fluid test chamber at varying initial volumes and baseline pressures

		Calculated Compliance (ΔV/ΔP) at the following set pressures (mmHg)					
↓ Decreasing Compliance	Initial Volume in Test Chamber (mL)	-10	0	10	20	50	100
	100	0.95	0.86	0.87	0.83	0.71	0.61
	200	0.76	0.72	0.81	0.75	0.63	0.59
	300	0.56	0.58	0.56	0.56	0.52	0.49
	400	0.52	0.47	0.47	0.44	0.43	0.44
	500	0.37	0.32	0.32	0.32	0.29	0.40
		→ Decreasing Compliance					

Progression of ICP with successive 1 mL boluses across different chamber volumes illustrated the inverse relationship between compliance and pressure change (Figure 15). Larger initial volumes produced more pronounced pressure increases per bolus, consistent with reduced compliance. The findings confirm that the compliance of the fluid chamber aligns with known physiological trends: compliance decreases as baseline pressure and chamber volume increase, which is consistent with established intracranial pressure-volume dynamics.

Clinical literature reports critical compliance values of approximately 0.9 mL/mmHg for pediatric patients (<16 years), 0.7 mL/mmHg for adults (16-60 years), and 0.6 mL/mmHg for older adults (>60 years). The test chamber achieved clinically relevant compliance when filled with 300 mL of water, indicating that this volume condition reproduces a compliance level similar to adult intracranial compliance. Adjustments to chamber volume can be made to approximate compliance profiles for different age groups or pathophysiological states.

This information provides a reference for understanding how changes in chamber pressure and volume relate to intracranial compliance. Compliance is influenced by multiple physiological factors such as parenchymal stiffness, CSF pathway obstruction, and hemorrhage location; these measurements illustrate how the IRRA*flow* system responds to bolus-induced pressure changes under controlled conditions.

Clinical importance: Understanding fluid chamber compliance (ΔV/ΔP) is relevant for tailoring treatment to patient-specific intracranial conditions. This study demonstrated compliance ranging from 0.86 mL/mmHg (0 mmHg, 100 mL) to 0.71 mL/mmHg (50 mmHg, 100 mL), aligning with adult clinical values of 0.7 mL/mmHg. In IVH, compliance data provide reference values that may inform irrigation settings to limit pressure fluctuations [2]. For SAH, compliance guidance may help contextualize expected pressure responses to fluid addition [1]. In ventriculitis, it provides information relevant to selecting appropriate drainage settings [15]. In cSDH, it may assist in determining drainage strategies that reduce abrupt pressure shifts [3].

These findings characterize how the system responds under varying simulated intracranial compliance conditions and may help inform clinical parameter selection.
